# Health Inequalities Among Elderly Type 2 Diabetes Mellitus Patients in Japan

**DOI:** 10.1089/pop.2019.0141

**Published:** 2020-06-03

**Authors:** Peng Jiang, Akira Babazono, Takako Fujita

**Affiliations:** ^1^ Department of Health Care Administration and Management, Graduate School of Medical Sciences, Faculty of Medical Sciences, Kyushu University, Fukuoka, Japan.; ^2^ Department of Health Sciences, Faculty of Medical Sciences, Kyushu University, Fukuoka, Japan.

**Keywords:** health inequality, diabetes mellitus, adherence, hospitalization, in-hospital death

## Abstract

The influence of socioeconomic status (SES) on health inequalities has received much attention worldwide. This study examined the effect of SES on the following older type 2 diabetes mellitus patient health outcomes: oral hypoglycemic agent (OHA) medication adherence (proportion of days covered, PDC), risk of hospitalization for diabetic macrovascular complications, and in-hospital death. A retrospective cohort design using 2013–2016 claims data was used. Subjects were 58,349 diabetes patients aged >74 years in 2013. Age, sex, residential area, and comorbidities were controlled for. Logistic regression was conducted to assess the effects of income on PDC; survival analysis was used to assess the effects on hospitalization and in-hospital death. Regressions were conducted separately by sex. Compared with the lowest income group, adjusted PDC odds ratios for medium- and high-income males, respectively, were 1.35 (95% CI: 1.27–1.43) and 1.41 (95% CI: 1.30–1.54); females: 1.17 (95% CI: 1.11–1.23) and 1.24 (95% CI: 1.13–1.35). Adjusted hazard ratios (AHRs) for male hospitalization were 0.88 (95% CI: 0.80–0.96) and 0.88 (95% CI: 0.79–0.99); females: 1.00 (95% CI: 0.93–1.07) and 0.95 (95% CI: 0.83–1.08). AHRs for male in-hospital death were 0.83 (95% CI: 0.75–0.91) and 0.62 (95% CI: 0.54–0.70); females: 0.94 (95% CI: 0.87–1.02) and 0.77 (95% CI: 0.65–0.92). Results revealed sex-specific health inequalities among older Japanese diabetes patients. Subjects with worse SES had significantly poorer OHA medication adherence (both sexes), higher hospitalization risk for diabetes complications (males), and higher in-hospital death risk (both sexes).

## Introduction

The relationship between socioeconomic status (SES) and health outcomes is a critical problem in social epidemiology.^1^ Some empirical studies have demonstrated an association between shorter life expectancy and SES-related health inequalities.

The population of Japan has one of the longest life expectancies in the world, and the increasingly aged population is a major problem. According to an estimate by the Japan Ministry of Internal Affairs and Communications Statistics Bureau, by May 1, 2018, there were 35.3 million (28.4%), 17.7 million (14.3%), and 5.6 million (4.5%) people older than ages 65, 75, and 85 years, respectively, in Japan.^2^ A National Health and Nutrition Survey conducted by the Japanese Ministry of Health, Labour and Welfare (MHLW) in 2016 showed that diabetes occurs frequently among the older population and has a substantial effect on quality of life (QOL); moreover, up to 10 million people are likely to have diabetes mellitus (DM), about 12.1% of the overall population of Japan.^3^ In addition, the proportion of both male and female DM patients older than age 60 years has been increasing.^3^

The incidence of diabetes complications is more frequent in older people.^3^ A combination of diabetes complications and frailty substantially affect the QOL of aging populations. The risk of developing diabetic macrovascular disease, including ischemic heart disease (IHD), stroke, and peripheral arterial disease (PAD), is higher in patients with diabetes than in patients without diabetes.^4,5^

Thus, daily control of blood sugar is important for older DM patients. Good medication adherence to oral hypoglycemic agents (OHAs) may reduce hospitalization for diabetic macrovascular complications.^6^ However, the aforementioned MHLW survey found that about 75% of people who are strongly suspected to have DM received medical treatment in Japan, and the proportion was higher for males than for females.^3^ Some studies have shown that nonadherence to OHAs may lead to subsequent hospitalization.^7^

Although a consensus is lacking, many studies have demonstrated that SES has a large impact on health outcomes in the older population.^7,8^ However, the effect of SES on medication adherence and diabetic macrovascular complications in DM patients in Japan is still unclear. Many Western studies suggest that differences between the sexes affect the association between SES and diabetes.^9–11^ However, only a few Asian studies have investigated the effect of sex on this association.^12,13^

Therefore, the study objective was to evaluate the effect of SES on older patients' medication adherence to OHAs and hospital admission for diabetic macrovascular complications by sex. The effects of SES on inpatient mortality also were assessed as a secondary outcome.

## Methods

### Data

This study used a retrospective cohort design. A health care claims database and master database for Fukuoka Prefecture Wide-Area Association of Latter-Stage Elderly Healthcare was used to obtain data for the period April 1, 2013, to March 31, 2016. The database is a component of Japan's social insurance system. People aged 75 years and older and those aged between 65 and 74 years are recognized by the Association as having a designated level of disability and are allowed to participate in this insurance plan. This health care claims database includes detailed data on diagnosis and treatment, such as *International Classification of Diseases, 10th Revision* (ICD-10) data, diagnosis date, drug prescriptions, and details of medical procedures. The master database includes information such as enrollers' demographic variables, and dates of enrollment and withdrawal. Most of this health care-insured population has sustainable long-term eligibility once they are enrolled; therefore, few study subjects were lost to follow-up because of moving to other prefectures or for other reasons. Most of these databases are computer administered and have a high penetration rate (98.6% as at April 2015). One reason for this is that the Japanese Health Insurance Claims Review & Reimbursement services are responsible for quality control of computer-administered claims databases.

The Institutional Review Board of Kyushu University (Clinical Bioethics Committee of the Graduate School of Healthcare Sciences, Kyushu University) approved this study.

### Definition of study subjects

First, patients with diabetes were defined as individuals who were diagnosed with diabetes (ICD-10 codes E10–E14) and prescribed oral antidiabetic agents or insulin in fiscal year 2013. Drugs were identified using the Japanese version of the National Health Insurance Drug List. Then, subjects younger than age 75 years were excluded from the study population because individuals younger than age 75 can only be enrolled in the health insurance plan if they have a disability; the inclusion of such individuals may have biased health outcomes data, as disability may affect hospital visits. Additionally, only patients prescribed OHAs in fiscal years 2014, 2015, and 2016 were included. Patients prescribed insulin also were excluded from the study, as detailed information about insulin treatment was not included in the administrative claims data, meaning that the ultimate subjects were type 2 diabetes patients.

### Definition of variables

OHA adherence was assessed using proportion of days covered (PDC) instead of medication possession ratio because of potential problems with overestimation and data availability with the latter. The denominator of OHA PDC was the number of days from April 1, 2014, until hospitalization admission for macrovascular complications or death. The numerator was the number of days covered by OHA prescription during the observation period. A PDC <80% was defined as nonadherence; this cutoff value has been used in many studies on chronic disease.^14,15^

The primary event was defined as hospitalization for diabetic macrovascular complications using ICD-10, including IHD, stroke, and PAD, as shown in Table 1. Number of days from beginning of observation until admission also were calculated. The secondary event was defined as hospital mortality; for this, the period from beginning of observation until hospital death was calculated.

**Table 1.** Patient Characteristics According to Sex

		Total	Female	Male	*P*
Age (years)	75–79	25,955	12,572	13,383	0.000
	80–84	18,626	9865	8761	
	≥85	13,768	9128	4640	
Income	low	17,145	12,599	4546	0.000
	medium	34,342	16,481	17,861	
	high	6862	2485	4377	
Charlson comorbidity index	0/2	9122	6036	3086	0.000
	3/4	15,247	8734	6513	
	≥5	33,980	16,795	17,185	
Region	Fukuoka/Itoshima	14,037	7482	6555	0.000
	Kasuya	2433	1281	1152	
	Munagata	1714	929	785	
	Chikushi	3690	1872	1818	
	Asakura	1285	691	594	
	Kurume	5492	3064	2428	
	Yame/Chikugo	1863	994	869	
	Ariake	3558	2005	1553	
	Idsuka	2775	1621	1154	
	Nogata	1750	959	791	
	Tagawa	2097	1172	925	
	Kitakyushu	14,988	8063	6925	
	Keichiku	2667	1432	1235	
Hypertension	No	7912	3696	4216	0.000
	Yes	50,437	27,869	22,568	
Hyperlipidemia	No	20,031	9378	10,653	0.000
	Yes	38,318	22,187	16,131	
Proportion of days covered	≤80%	24,107	13,182	10,925	0.017
	>80%	34,242	18,383	15,859	
Acute myocardial infarction	No	57,916	31,348	26,568	0.095
	Yes	433	217	216	
Ischemic heart disease	No	55,252	30,070	25,182	0.000
	Yes	3097	1495	1602	
Hemorrhagic stroke	No	56,522	30,608	25,914	0.135
	Yes	1827	957	870	
Ischemic stroke	No	55,980	30,441	25,539	0.000
	Yes	2369	1124	1245	
Peripheral arterial disease	No	57,378	31,107	26,271	0.000
	Yes	971	458	513	
Inpatient death	No	52,822	28,962	23,860	0.000
	Yes	5527	2603	2924	

Income group was used as the SES indicator because no detailed income data were available. Based on the insurance policy, the income group for each enroller was determined by their income level, reflecting each enroller's SES. The income group was divided into 3 groups according to the insurance policy: lowest, medium, and highest income groups. The lowest income and medium-income groups had a co-payment of 10%. The highest-income group, who had a co-payment of 30%, was defined as those whose incomes were comparable to the current workforce. Age was categorized into 3 groups: 75–79 years, 80–84 years, and 85 years or older. The Charlson comorbidity index (CCI) for all conditions and ICD-10 codes were used to assess medical complications, except for mild diabetes and diabetes with complications. CCI was categorized into 3 groups: 0–2, and 3–4, or ≥5. Residential area also was adjusted for in the regression model, using the Japan Secondary Healthcare Area, which divides Fukuoka Prefecture into 13 subareas. Furthermore, other lifestyle-related diseases (hypertension [I10] and hyperlipidemia [E78.0–78.5]) also were controlled for as confounding factors using ICD-10 codes from medical claims data during the study period.

### Statistical analysis

Patient characteristics were described by frequency counts and proportions for categorical variables, and Pearson χ^2^ tests were conducted to analyze categorical variables by sex.

A multivariate logistic regression model was used to assess the effect of income on OHA PDC, because PDC is a binary variable. Adjusted odds ratios (AORs) and 95% confidence intervals (95% CIs) were estimated for different income groups. In this model, residential area, age, CCI, hypertension, and hyperlipidemia were set as independent variables.

For hospitalization of macrovascular disease and hospital mortality, a survival model was used to estimate the effect of SES. Time from beginning of observation to first incidence of admission for macrovascular hospitalization was analyzed as the time-to-event. The diagnosis date recorded in the electronic health record, or the censoring at the final event if the events were not observed, was used as the end point. Kaplan-Meier survival curves were produced, and the log-rank test and the Cox proportional hazard model were used to estimate hazard ratios and 95% CIs. In the Cox proportional hazard model, residential area, age group, CCI, hypertension, and hyperlipidemia were adjusted as covariates. For all models, the significance level was set at *P* < 0.05, and data extraction and analyses were conducted using Microsoft SQL Server 2014 (Microsoft Coporation, Redmond, WA) and Stata Statistical Software, release 14 (StataCorp LLC, College Station, TX).

## Results

### Descriptive statistics

A total of 58,349 patients were identified, and 54.2% were female. Table 1 shows subject characteristics by sex. The median ages for males and females were 80 years and 81 years, respectively. Significantly more males than females had high incomes. Significantly more females than males had hypertension and hyperlipidemia, but the CCI of females was lower. For outcome variables, the medium PDC values for males and females were 90.2% and 89.4%, respectively, and the proportion of subjects with PDC >80 was slightly but significantly higher in males than in females.

### Results for OHA PDC

For males, compared with subjects with the lowest incomes, the AOR for subjects with medium incomes was 1.35 (95% CI: 1.27–1.43, *P* < 0.01) and the AOR of those with the highest incomes was 1.41 (95% CI: 1.30–1.54, *P* < 0.01). For females, compared with subjects with the lowest incomes, the AOR of subjects with medium incomes was 1.17 (95% CI: 1.11–1.23, *P* < 0.01) and the AOR of subjects with the highest incomes was 1.24 (95% CI: 1.13–1.35, *P* < 0.01). The magnitude of the effect of income among males was obviously greater than among females.

### Results of the survival analysis for hospitalization and in-hospital death

**FIG. 1.**
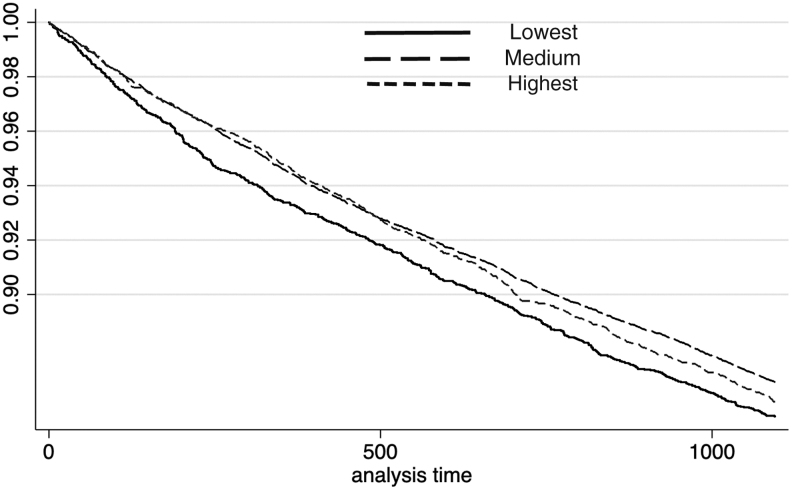
Macrovascular hospitalization for males.

**FIG. 2.**
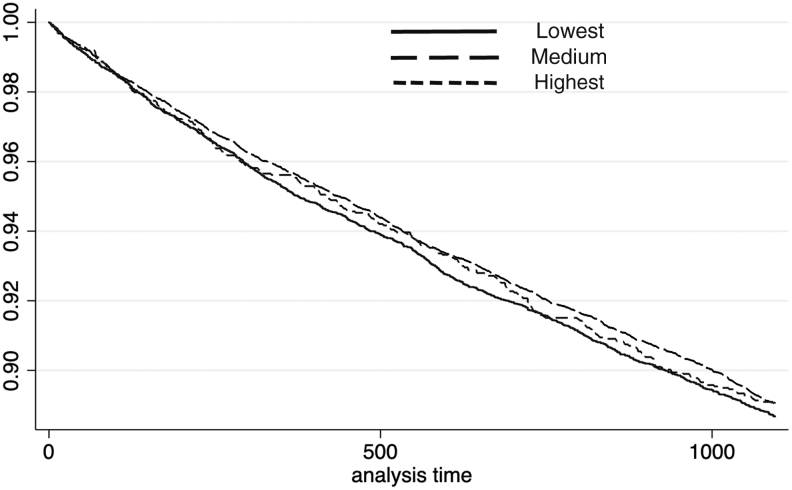
Macrovascular hospitalization for females.

For macrovascular hospitalizations among males, the *P* value of the log-rank test for different income categories was 0.05 (χ^2^ = 5.97). The Cox model indicated that, compared with those with the lowest incomes, the adjusted hazard ratio (AHR) of subjects with medium incomes was 0.88 (95% CI: 0.80–0.96, *P* < 0.01) and the AHR of subjects with the highest incomes was 0.88 (95% CI: 0.79–0.99, *P* = 0.03). However, there were no significant differences in AHR among females within different income groups. The *P* value of the log-rank test was 0.55 (χ^2^ = 1.20). Compared with those with the lowest incomes, the AHR of females with medium incomes was 1.00 (95% CI: 0.93–1.07, *P* = 0.98), and the AHR of females with the highest incomes was 0.95 (95% CI: 0.83–1.08, *P* = 0.44). Kaplan-Meier curves for macrovascular hospitalizations among different income groups are shown in Figure 1 and Figure 2.

**FIG. 3.**
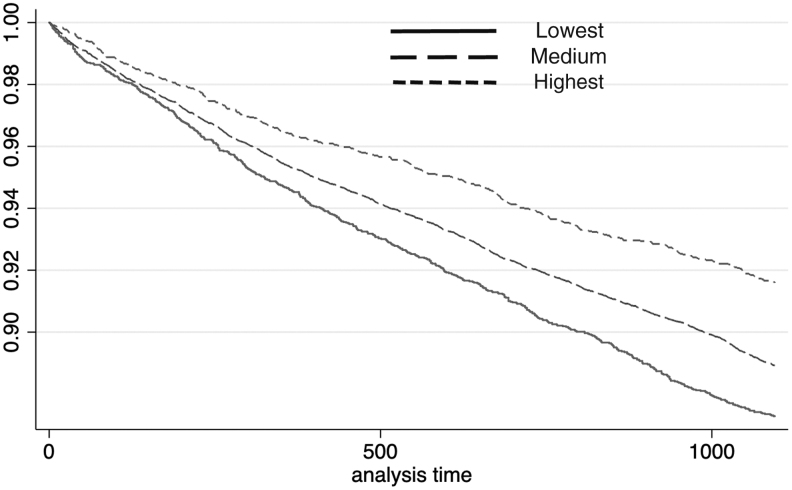
In-hospital death for males.

**FIG. 4.**
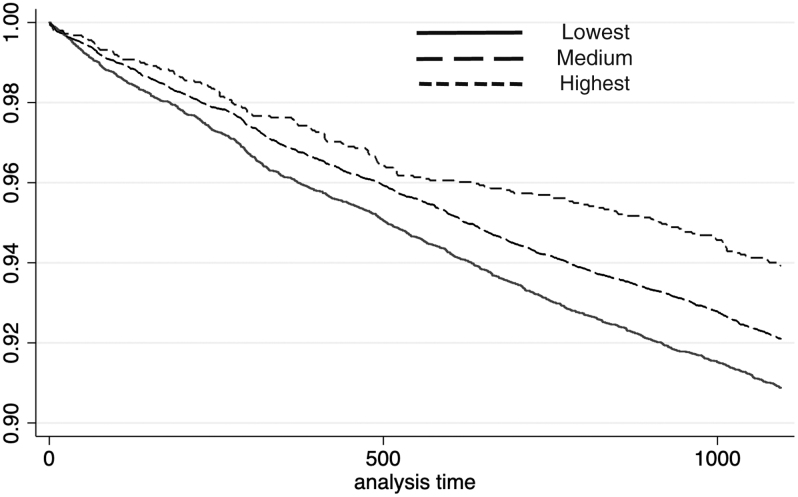
In-hospital death for females.

For the in-hospital death event, the *P* value of the log-rank test for males in different income categories was smaller than 0.01 (χ^2^ = 43.97). Compared with subjects with the lowest incomes, the AHR of males with medium incomes was 0.83 (95% CI: 0.75–0.91, *P* < 0.01) and the AHR of males with the highest incomes was 0.62 (95% CI: 0.54–0.70, *P* < 0.01). For females, the *P* value of the log-rank test was smaller than 0.01 (χ^2^ = 30.89). Compared with those with the lowest incomes, the AHR of female subjects with medium incomes was 0.94 (95% CI: 0.87–1.02, *P* = 0.15), which suggested that there was no significant between-group difference for in-hospital death hazard. The AHR of females with the highest incomes was 0.77 (95% CI: 0.65–0.92, *P* < 0.01). Kaplan-Meier curves for in-hospital death among different income groups are shown in Figure 3 and Figure 4.

## Discussion

SES determines 3 major aspects of health: health care, environmental exposure, and health behavior.^16^ Findings from this study showed that lower income level, which is an important indicator of poorer SES, had a significantly negative effect on older DM patients' health outcomes of OHA medication adherence, hospitalization for diabetic macrovascular complications, and, ultimately, in-hospital mortality.

OHA medication adherence is a measure of patient health behavior. This study found that although males had slightly better adherence than females, both sexes exhibited a significant decrease in PDC according to lower income level, indicating that worse SES had a significantly negative effect on older DM patients' OHA medication adherence. These results are consistent with many previous findings.^17–19^ Nonadherence can be caused by the burden of increased expenditure.^14^ Moreover, the odds ratios in this study showed that the adherence decrease for males was greater than for females, indicating that there might be a correlation between SES and sex when discussing health disparities.

Many previous studies have demonstrated income inequality in hospitalization,^20^ not only in DM patients but also in the general population.^21,22^ The present findings are consistent with those of previous studies. This study found that older male DM patients with medium and lowest incomes were at significantly greater risk of hospitalization for diabetic macrovascular complications than those at the highest income level. The trend was similar for females; however, there was a significant difference in hospitalization risk only for subjects with the lowest and the highest incomes, which was consistent with the results of the log-rank test.

Several previous studies have reported that better medication adherence is associated with the lowest hospitalization rates among patients with DM and other lifestyle-related diseases.^23^ There also is evidence that lower adherence to DM medication is associated with higher risk of subsequent hospitalization.^6,7^ Several studies have demonstrated an association between poor OHA adherence and metabolic control.^24^ In addition, glucose control is important for vascular outcome management in DM patients. One recent meta-analysis suggested that lowering glucose is important to prevent long-term microvascular complications in adults with type 2 diabetes.^25^ Given the link between poor adherence and lower SES, there may be an association between OHA medication adherence and macrovascular hospitalization.

Regarding the risk of in-hospital death, many studies have suggested that lower income is associated with a higher risk of in-hospital mortality.^26,27^ Present study findings show that both male and female older DM patients with higher incomes had significantly reduced inpatient death. However, the effect of income level on in-hospital death may be multifactorial. SES factors such as housing conditions, poor nutrition, and lack of access to preventive services may have negative effects on health in low-SES populations.^28^ Although these populations tend to have higher rates of chronic diseases and other comorbidities, they have poorer baseline health status than high SES populations.^29,30^ As a result, multifaceted dimensions of poor SES may combine to have a negative effect on disparities for in-hospital deaths.

In this study, age, residential area, and comorbidities were adjusted for to examine the effect of SES. Age was found to be significantly associated with OHA nonadherence, hospitalization admission, and in-hospital death in both males and females, indicating that older DM patients had worse OHA medication adherence. However, previous research has failed to show a consistent association between age and nonadherence in DM patients. Some studies have found no association,^31,32^ whereas others show that medication adherence improves with age.^33^ Although previous studies have shown the same association between age and nonadherence as was found in the present study,^34^ especially for older DM patients, nonadherence may be associated with aging because of higher prevalence of cognitive impairment in older individuals.^35,36^ One recent study suggests that cognitive impairment may decrease medication adherence in older chronic disease patients without dementia.^37^

Regarding the effect of comorbidities, both male and female subjects showed a trend for higher CCI to be associated with nonadherence and a higher risk of hospitalization and in-hospital death. Subjects with higher CCI scores may have been prescribed more medication than those with lower CCI scores. Some studies have suggested that prescribing and dispensing patterns may contribute to the accumulation of unwanted and unused medications,^38^ and that polypharmacy may be linked to a higher risk of nonadherence^39,40^; however, data about polypharmacy were not available in the study. Additionally, in this study DM patients with hypertension or hyperlipidemia had better adherence than those without these conditions.

### Limitations

SES is usually conceptualized in terms of multidimensional indicators, such as income, education level, occupation, and housing conditions. However, only income was examined in this study because of data availability. This study emphasized lower medication adherence as the main factor that led to poorer diabetes outcomes in the lower SES group; however, other factors may matter, such as nutrition and exercise management. Nevertheless, the information was not available. Another limitation is that although almost all residents in Fukuoka Prefecture were included, individuals from countries other than Japan were not included; therefore, the data may not be generalizable to other populations and ethnic groups. Furthermore, the study used a retrospective design and data from a claims database, which did not include information about DM severity (although diabetes complications were assessed using CCI). In claims databases, input and coding errors also are possible, although these are likely to be random errors with little influence on the statistical inferences drawn.

Additional prospective studies involving diverse ethnic groups and multidimensional SES indicators are required to obtain higher quality evidence of health disparities among older DM patients.

## Conclusion

There are health inequalities among older DM patients in Japan. The present study showed that worse SES was associated with significantly poorer OHA medication adherence for both sexes, higher risks of hospitalization for diabetic macrovascular complications for males, and higher risk of in-hospital death for both sexes. The authors suggest that lower OHA medication adherence related to lower income may increase the risk of hospitalization for diabetic macrovascular and in-hospital mortality. This effect is sex-specific; that is, low-SES males had significantly greater morbidity and hospital mortality, whereas low-SES females only showed significantly greater hospital mortality. Therefore, insurer strategies that motivate beneficiaries with DM to improve medication adherence may be effective in reducing the risk of hospitalization for diabetic macrovascular complications and in-hospital mortality, thus reducing unnecessary medical expenditure.
